# *RAD50* Loss of Function Variants in the Zinc Hook Domain Associated with Higher Risk of Familial Esophageal Squamous Cell Carcinoma

**DOI:** 10.3390/cancers13184715

**Published:** 2021-09-21

**Authors:** Josephine Mun Yee Ko, Shiu Yeung Lam, Lvwen Ning, Annie Wai Yeeng Chai, Lisa Chan Lei, Sheyne Sta Ana Choi, Carissa Wing Yan Wong, Maria Li Lung

**Affiliations:** Department of Clinical Oncology, The University of Hong Kong, Hong Kong, China; mrlam510@gmail.com (S.Y.L.); u3003773@connect.hku.hk (L.N.); waiyeeng@connect.hku.hk (A.W.Y.C.); u3004461@connect.hku.hk (L.C.L.); ssachoi2@connect.hku.hk (S.S.A.C.); wwyc23@hku.hk (C.W.Y.W.)

**Keywords:** familial ESCC, *RAD50*, DNA repair, loss of function mutation, NGS, Chinese, genetic susceptibility, zinc hook domain, ATR, synthetic lethality, CHK1 inhibitor, replication stress

## Abstract

**Simple Summary:**

Two deleterious *RAD50* loss-of-function germline mutations were identified from the blood DNA of a cohort of 3289 Henan individuals by next-generation sequencing. These rare loss-of-function *RAD50* variants were associated with a substantial increased risk of familial esophageal squamous cell carcinoma in high-risk Northern China. A functional study suggested that the *RAD50* mutations may affect DNA repair and cell survival upon replication stress. Our preliminary functional study provided novel insight and the potential clinical implication that patients with heterozygous *RAD50*^L1264F^ and *RAD50*^Q672X^ status may have a potential synthetic lethal therapeutic option with CHK1 inhibitors. Further study is warranted for validation of the implicated genetic susceptibility role of the *RAD50* Zinc Hook mutants.

**Abstract:**

Unbiased whole-exome sequencing approaches in familial esophageal squamous cell carcinoma (ESCC) initially prioritized *RAD50* as a candidate cancer predisposition gene. The combined study with 3289 Henan individuals from Northern China identified two pathogenic *RAD50* protein truncation variants, p.Q672X and a recurrent p.K722fs variant at the zinc hook domain significantly conferring increased familial ESCC risk. Effects of ~10-fold higher familial ESCC risk were observed, when compared to East Asians from the gnomAD database. Functional characterization suggested that the *RAD50*^Q672X^ mutation contributes a dominant-negative effect in DNA repair of double-stranded breaks. Overexpression of the *RAD50*^Q672X^ and *RAD50*^L1264F^ missense mutation also sensitized cell death upon replication stress stimuli induced by formaldehyde treatment and the CHK1 inhibitor, AZD7762. Our study suggested the novel insight of the potential for synthetic lethal therapeutic options for *RAD50*^Q672X^ and the East-Asian-specific *RAD50*^L1264F^ variants and CHK1 inhibitors. Our study also suggested the association of *RAD50* LOF variants in the zinc hook domain with a higher risk of familial ESCC in Chinese.

## 1. Introduction

Esophageal cancer (EC) is ranked ninth among cancers in global incidence with 572,034 newly diagnosed cases and a dismal 5 year survival rate of less than 5% [1]. EC poses an immense health risk worldwide [1]. The distribution of histological subtypes of EC displays geographical correlations. In Eastern countries where EC is prevalent, such as China, squamous cell carcinoma (SCC) is the predominant histological EC subtype, as opposed to adenocarcinoma (AC) being the more common form of EC in the West [2]. The epidemiological risk factors of esophageal squamous cell carcinoma (ESCC) differ between the high-risk China and moderate-risk Japanese regions [2]. Although genome-wide association study (GWAS) provided genetic evidence for the modest effect of common variants and ESCC risk, the current understanding of the genetic pathogenesis and the genetic risk of rare deleterious variants with large effects on ESCC is still poor [3,4,5]. ESCC occurs with an extraordinarily high incidence (>100/100,000 for countries residing in the “Asian cancer belt” compared to <10/100,000 in Western developed countries, e.g., USA and France), with dismal survival in Northern China, highlighting the unmet need for early detection to improve patient survival [6]. Great geographical variation of incidence of about a 21-fold difference occurred between the highest-incidence countries in Eastern Asia and the lowest-incidence countries in Western Africa [6]. The management of the disease remains challenging, especially in high-risk areas such as Henan, Shanxi, and the Tai-Hang mountain area, because patients present at a late stage due to the difficulty of the expensive and invasive endoscopic screening. Endoscopy with Lugol’s iodine staining, known as chromoendoscopy, is utilized as a population-level screening of premalignant and early-stage malignant lesions in some high-risk areas including Northern China, such as Linxian province [7,8,9]. Chromoendoscopy is also used in screening high-risk alcoholic individuals in Japan [10], in East Asia and Brazil [11], and patients with head and neck or tracheobronchial cancer in France [12]. Familial aggregation in the high-risk region implicates genetic factors as playing an etiological role [13,14]. Hence, we performed a next-generation sequencing (NGS) study utilizing whole-exome sequencing (WES) and deep target capture sequencing of an oncology panel of 598 genes with ESCC patients from Henan, a hotspot of ESCC incidence in China, to understand the genetic basis for this cancer. Our earlier study identified multiple candidate cancer predisposition genes including genes involved in DNA repair (*BRCA2*, *POLQ*, and *MSH2*), inflammation (*TTC39B*), and angiogenesis (*KDR*), significantly associated with ESCC risk [15]. *RAD50* was prioritized by applying a loss-of-function (LOF) filtering strategy in our initial effort as one of the top candidate genes for further data mining to reveal its role in genetic susceptibility in ESCC development in the high-risk region.

*RAD50* mapping to 5q31 encodes a 153 kDa protein, which is a part of the MRE11-RAD50-NBS1 (MRN) complex. The MRN complex is important for the maintenance of the genomic integrity to prevent neoplastic transformation by orchestrating the DNA damage response to DSBs and stalled replication fork, telomere maintenance, and immune responses upon viral infection [16]. It exerts its effect in various stages of cellular DNA damage response (DDR) including DSB sensing, DDR cascade initiation, and repair pathway decisions to maintain genome stability [17,18,19]. Notably, the MRN complex is responsible for the activation of ataxia-telangiectasia mutated (ATM), the apical signaling kinase that initiates cell cycle arrest, and the vast downstream network of effectors in DDR to DSBs [20,21].

Human RAD50 deficiency is much less common compared to *NBN* (*NBS1*) or *MRE11A* mutations of the MRN complex, and to date, is a poorly understood disorder. Biallelic *RAD50* LOF mutations were reported in only two patients who presented with a Nijmegen breakage syndrome (NBS)-like but distinctive phenotype with key clinical features including facial dysmorphisms, congenital microcephaly, prenatal and postnatal growth retardation, short stature, mild intellectual disability, and radioresistant DNA synthesis [22,23,24]. The third patient with biallelic *RAD50* mutations with a LOF null allele and a separation-of-function allele (non-frameshift deletion at amino acid 1035, *RAD50*^E1035^^Δ^) had bone marrow failure and developmental defects [25]. Cells from patients with these *RAD50* null alleles have a low level of RAD50 protein and exhibit chromosomal instability and radioresistant DNA synthesis [22,23,24,25]. Unlike the NBS, the patients with biallelic LOF *RAD50* mutations had no immunodeficiency. RAD50 haploinsufficiency observed in T-lymphocytes of Finnish breast cancer patients carrying heterozygous LOF mutations such as the Finnish founder mutation *RAD50* 687delT resulted in increased genomic instability [26].

The genetic predisposition role of *RAD50* in inherited breast cancer [26,27] has been documented, while its role in breast cancer is still controversial [23,24,28]. Damiola et al. reported the rare missense and protein-truncation germline mutations located in the key functional domains in the MRN complex associated with breast cancer risk for a cohort of 1313 early-onset breast cancer patients [28]. However, *RAD50* germline mutations were not associated with breast cancer risk in a large cohort of 7657 unselected Chinese breast cancer patients [29]. The *RAD50* pathogenic mutations in breast cancer such as the Finnish founder mutation *RAD50* 687delT may be population specific [26]. These earlier contradictory observations support the notion that rare deleterious missense or LOF mutations in key functional domains of *RAD50* may associate with breast cancer in an ethnicity specific or early-onset manner. These earlier MRN complex studies supported our current ESCC study to examine whether rare mutations in the key functional domain of *RAD50* predispose individuals to develop ESCC in Northern Chinese in high-risk regions. The genetic susceptibility role of *RAD50* in familial ESCC is largely unknown. Germline *RAD50* mutations may contribute to hereditary cancer in human as hypomorphic *RAD50* mutants exhibited cancer predisposition in mice [30]. Hence, the current study aimed to utilize NGS approaches to study the role of *RAD50* in familial ESCC risk in the high-risk region from China. The current study showed by massive parallel sequencing that rare germline LOF variants of *RAD50* (*RAD50*^Q672X^ and *RAD50*^K722fs^) in the zinc hook domain were significantly elevated in familial ESCC cases with large effects.

The potential clinical implication of therapeutically targeting *RAD50* in cancers has been demonstrated by dominant negative disruption of *RAD50*, conferring sensitization to platinum-based chemotherapy in squamous cell carcinoma [31]. Hence, we also aimed to perform functional characterization of the dominant negative effect of *RAD50* mutants with regards to DNA damage stimuli inducing DSBs and various genotoxic agents.

## 2. Materials and Methods

The current study included a total of 3289 Henan Chinese participants (1044 familial and 1074 sporadic ESCC, and 1171 controls) collected during 2001–2014 from Northern China high-risk Linxian and Anyang counties from Linzhou Center Hospital (Henan, China) and Yaocun Esophageal Cancer Hospital (Henan, China) provided by Lidong Wang (Zhengzhou University) for an earlier study [15]. In the discovery phase, WES was performed in 186 family history-positive (FH+) ESCC with two generations and ≥2 family members diagnosed with ESCC including proband. In the validation phase, target capture sequencing was performed in 858 FH+ ESCC (defined as ≥2 family members diagnosis with ESCC including proband in one generation), 1074 sporadic ESCC, and 1171 geographically matched controls without ESCC). In the combined analysis, the 1044 familial ESCC included the 186 FH+ ESCC and 858 FH+ ESCC. The gnomAD database contains variations in ~141,000 individuals resulting from the aggregation of exomes and genomes from case-control sequencing studies of common adult-onset diseases including cardiovascular disease, type 2 diabetes, and psychiatric disorders [32]. The East Asians (9977) in genomAD contain 76 Japanese, 1909 Korean, and 7992 other East Asians. All populations in genomAD (141,335) contain East Asian (9977), non-Finnish European (64,603), Finnish (12,562), Latino/Admixed American (17,720), South Asian (15,308), African/African American (12,487), Ashkenazi Jewish (5185), and others (3614) [32]. The *RAD50* variants information from genome build GRCh37/hg19 in dataset genomAD v2.1.1 was exported by input of the gene name “*RAD50*” into the gnomAD browser from the genome Aggregation Database (gnomAD; https://gnomad.broadinstitute.org, accessed on 5 June 2020). The details of all study populations are summarized in Table 1. Approval for use of human blood and/or information was obtained from the Committee for Ethical Review of Research Involving Human Subjects at Zhengzhou University (Henan, China). The study was conducted according to the Declaration of Helsinki principles. Informed written consent was obtained from all participants.

### 2.1. WES, Target Capture Sequencing, and Bioinformatics Analysis

Blood DNAs were extracted by QIAamp DNA blood mini kit (QIAGEN, Germany). Details of library preparation with the KAPA HTP Library Prep Kit, exome sequencing, and target capture with NimbleGen SeqCap EZ capture kits (Roche, Switzerland) were as previously published [15]. The data from 3289 individuals were processed using the analysis pipeline following the GATK guideline, as previously described [15,33]. In brief, raw fastq reads were cleaned and then mapped to the human reference genome hg19 using the Burrows–Wheeler aligner (BWA). PCR duplicates are marked using Picard. InDels realignment and variants recalibration were performed using GATK. ANNOVAR was used for functional annotation of variants. Combined annotation-dependent depletion (CADD) score was used to assess damage effects of variants of unknown significance [34]. The rare variants of unknown significance with CADD score of ≥25 (version1.4) are considered deleterious variants and listed in Supplementary Table S1.

### 2.2. Statistical Analysis

In NGS analysis, for the rare *RAD50* LOF variant, the Fisher’s exact test (two-tailed) was used for calculation of the odds ratio (OR) between cases and control using a Python package SciPy and a *p* < 0.05 was considered statistically significant. Student’s t test was used in statistical analyses in the cell viability and colony formation assays. A *p* value < 0.05 was considered as statistically significant. The error bars in the figures represent the standard error mean.

### 2.3. Cell Culture

KYSE150, an ESCC cell line derived from poorly differentiated primary tumor, and U2OS, an osteosarcoma cell line, were used in the in vitro studies. KYSE150 was cultured with RPMI medium as previously described [35]. U2OS was cultured using Dulbecco’s modified Eagle’s medium (DMEM) with 10% fetal bovine serum and 1% penicillin and streptomycin.

### 2.4. RAD50 Constructs, Lentiviral Preparation, and Transduction

Functional knockout of human *RAD50* and expression of *RAD50*^WT^ and mutants were performed via lentiviral transduction using the LentiCRISPRv2 plasmid, as previously described [36,37]. Functional knockout of *RAD50* in cell lines was performed with the sgRNA sequences: LacZ Fwd-CTCTGGCTAACGGTACGCGTA, Rev-TACGCGTACCGTTAGCCAGAG; S1-GTTCCGCGTTACATAACTTA, Rev-TAAGTTATGTAACGCGGAAC as control, and ex2 (*RAD50* KO) Fwd-CACCGTACATTTGTACACGATCCCA, Rev-AAACTGGGATCGTGTACAAATGTAC; ex10 (*RAD50* KO) Fwd-CACCGCTAGGAACGTGAGTTAAGCA, Rev-AAACTGCTTAACTCACGTTCCTAGC as *RAD50* KO sgRNAs. The subcloning of respective sgRNAs into the plasmid was performed as previously described. Expression of *RAD50* wildtype and mutant constructs was performed using the pLVX-EF1a-Puro plasmid. Full-length CDS of *RAD50* was isolated from pTP11 (a gift from Prof. Tanya Paull, University of Texas at Austin, Texas, USA) and subcloned into pLVX-EF1a-Puro. Mutants of *RAD50* were generated by the GeneArt^®^ Site-directed Mutagenesis Kit (Invitrogen). Mutagenesis primers used are as follows: L1264F Fwd-CAGCGTAACTTCCAGTTTCTGGTAATCACTC, Rev-GAGTGATTACCAGAAACTGGAAGTTACGCTG, and Q672X Fwd-TCCCAGTTCATTACTTAGCTAACAGACGAAA, Rev- TTTCGTCTGTTAGCTAAGTAATGAACTGGGA.

### 2.5. Western Blotting

Western blots were performed with primary antibodies anti-RAD50 (GTX70228, Genetex, Irvine, CA, USA), anti-MRE11 (GTX70212, Genetex, Irvine, CA, USA), anti-NBS1 (GTX70224, Genetex, Irvine, CA, USA), anti-6xHIS (#12698, Cell Signaling Technology, Danvers, MA, USA), anti-α-tubulin (GTX112141, Genetex, Irvine, CA, USA), and anti-P84 (GTX70220, Genetex, Irvine, CA, USA), as described previously [36,37].

### 2.6. Ionizing Radiation and Immunofluorescence Staining

Cells received the ionizing radiation treatment at a cumulative dose of 10Gy by Gamma Irradiator MDS Gammacell 3000 Elan. Immunofluorescence staining was performed as previously described [37]. Briefly, cells were fixed with 4% paraformaldehyde and permeabilized with 0.1% Triton X-100 in PBS. Primary antibody anti-γH2AX (#9718, Cell Signaling Technology, Danvers, MA, USA) was incubated overnight at 4 °C and with Alexa-488 Fluor^®^ secondary antibodies for 1 h at room temperature, in dark. Cells were visualized using Nikon Ti2-E Widefield Imaging System and image analyzed with Image J. For foci quantification, cells with >10 foci were counted. At least 1000 cells were counted for each sample.

### 2.7. Cell Viability and Colony Formation Assays

Cell viability was measured by the 3-(4,5-dimethylthiazol-2-yl)-2,5-diphenyl-tetrazolium bromide (MTT) assay, as reported previously [38]. In brief, 1 × 10^4^–2.5 × 10^4^ cells were seeded in triplicates in each well of a 96-well plate. Cells were treated for cisplatin at concentration range from 10 to 1000 nM, formaldehyde at concentration range from 10 to 800 µM, and AZD7762 at concentration range from 5 to 100 nM. Colony formation assay was performed as previously described [38]. In brief, fixation and staining with Giemsa, colonies were counted in DMSO and AZD7762 (20 nM) treatment after one week. All experiments were repeated twice.

## 3. Results

### 3.1. WES Analysis Prioritizes RAD50 as Top Candidate Cancer Predisposition Gene (CPG) for Familial ESCC

By combining the enrichment of genetic component strategy and unbiased WES approach, we performed NGS analysis for 186 FH+ ESCC individuals [15]. After WES data analysis in the discovery phase applied a LOF filtering strategy, *RAD50* was prioritized as the top candidate CPG for validation in a larger cohort (Table 2). The prevalence of *RAD50* LOF variants in Henan FH+ ESCC patients (4/372, 1.1%) was significantly more frequent compared to that from the East Asian population in the gnomAD public database (30/19,954, 0.15%) (OR = 7.22, *p* = 3.3 × 10^−3^). Two out of the four *RAD50* LOF variants (c.C2014T:p.Q672X and c.2165_2166insT:p.K722fs) are located at the zinc hook domain at amino acids 635-734 [28].

### 3.2. Two RAD50 LOF Variants at the Zinc Hook Domain Associate with Increased Risk of Familial ESCC

**Figure 1:**
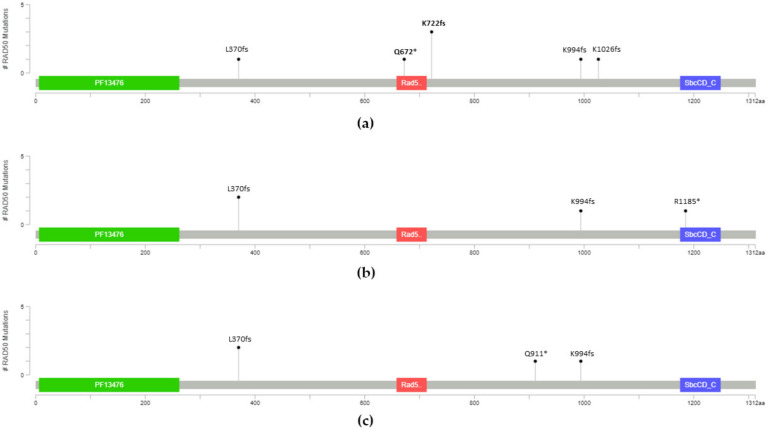
Lollipop schematic diagram of *RAD50* LOF mutation distribution in (**a**) familial ESCC cases, (**b**) sporadic ESCC cases, and (**c**) controls from Henan. * stands for stop gain mutation.

We performed target whole *RAD50* sequencing in a larger cohort of 3103 ESCC patients including additional 858 FH+ ESCC and 1074 sporadic ESCC, and 1171 controls (Table 1). For the validation cohort, we detected 3/1716 (0.17%) *RAD50* LOF variants in FH+ ESCC, 4/2148 (0.19%) in sporadic ESCC and 4/2342 (0.17%) in the controls (Table 2). Overall, a total of seven pathogenic *RAD50* LOF variants were detected at exons 8, 13, 17, 19, and 23 in 15 individuals (Table 2) consisting of seven familial ESCC (Figure 1a), four sporadic ESCC patients (Figure 1b), and four control individuals (Figure 1c). Figure 1 shows the lollipop schematic diagram of seven *RAD50* LOF variants. Four of the fifteen carriers (26.7%, 4/15) had LOF *RAD50* variants mapping to exon 13 containing the zinc hook domain. We observed a trend of higher frequency of *RAD50* zinc hook LOF mutations in FH+ ESCC, 4/2088 (0.19%) compared to that of sporadic ESCC, 0/2148 (0%) (OR inf, *p* = 0.059) (Table 3). Owing to the very rare minor allele frequencies (MAFs) of *RAD50*^Q672X^ (0.045%) and *RAD50*^L1264F^ (0.14%) in familial ESCC, the *p* value may be compromised. Based on the assumption that familial ESCC patients may inherit a defective copy of the CPG from one of their parents, while sporadic ESCC patients may acquire somatic mutations during their lifetime and the observation of the absence of the *RAD50* zinc hook LOF mutations in both sporadic ESCC and control cohorts, they were combined and defined as the nonfamilial control population (Table 1) to estimate the risk of familial ESCC patients. In the familial ESCC patients, the frequency of two pathogenic variants, p.Q672X and the other recurrent p.K722fs variant (4/2088, 0.19%), is statistically higher than that in the nonfamilial controls (0/4490, 0%) (OR inf, *p* = 0.010) (Table 3). An increased risk with large effect in familial ESCC patients carrying these two pathogenic *RAD50* LOF variants is also observed, when compared to East Asians from the gnomAD database (4/19,954, 0.02%) (OR 9.57, *p* = 4.1 × 10^−3^) and all populations from gnomAD (5/282,670, 0.0018%) (OR 108.51, *p* = 3.5 × 10^−7^). p.Q672X is a very rare truncating *RAD50* mutation disrupting the zinc hook. It is absent in the sporadic ESCC and Henan noncancer control cohorts, as well as the noncancer East Asian population from the gnomAD (MAF 0%, 0/9,977) (OR inf, *p* = 0.095) but observed with more than 100-fold higher frequency in familial ESCC risk (0.045%, 1/2088) compared to all populations from genomAD (MAF 0.00035%, 1/282,670) (OR 135.44, *p* = 0.015). Although the rarity of p.Q672X may raise concern for its significance, it is considered as a pathogenic germline mutation previously reported in two patients with hereditary cancer predisposing syndrome and one breast carcinoma patient in the ClinVar database [39]. We provide further functional characterization and suggest that this variant contributes to a dominant negative effect in DNA repair. The recurrent p.K722fs was more frequent in familial ESCC (3/2088, 0.14%) compared to sporadic ESCC and control (0/4490, 0%) (OR inf, *p* = 0.032, Table 3). p.K722fs only occurs in the East Asian population of gnomAD with minor allele frequency (MAF) 0.02% (4/19,945) (OR = 7.18, *p* = 0.022) but is absent in other populations of gnomAD including African, Latino, Jewish, European, and South Asian (0.0014%, 4/282,670) (OR 101.73, *p* = 1.3 × 10^−5^). The ClinVar database also classified this recurrent zinc hook mutant at p.K722fs as a germline pathogenic mutation in two patients with hereditary cancer predisposing syndrome [40].

The estimated risk of familial ESCC carrying LOF *RAD50* variants (7/2088, 0.34%) was moderately increased compared to Henan sporadic ESCC cases and controls (8/4490, 0.18%), although this was not statistically significant (OR 1.88, *p* = 0.33). A similar trend of moderately increased risk (30/19,945, 0.15%) (OR 2.22, *p* = 0.092) was observed, when compared to East Asians from the gnomAD database and all populations from gnomAD (427/282,670, 0.17%) (OR 2.22, *p* = 0.062, Table 3).

### 3.3. Sanger Sequencing Validation of RAD50 Germline Variants

**Figure 2:**
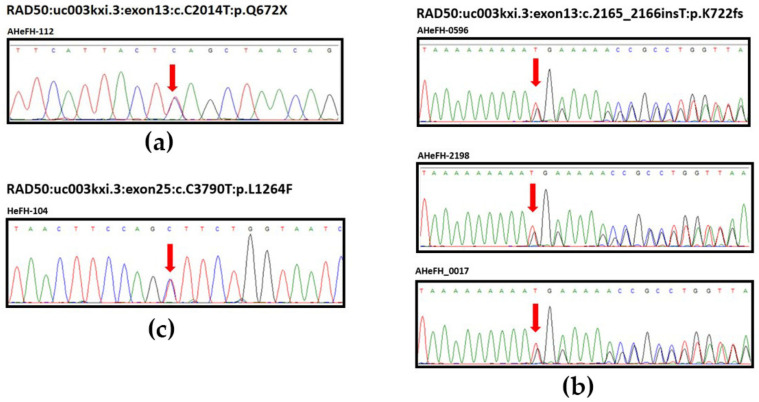
Sanger sequencing validation of *RAD50* LOF germline mutations, (**a**) c.C2014T:p.Q672X, (**b**) c.2165_2166insT:p.K722fs, and (**c**) c.C3790T:p.L1264F in five familial ESCC patients.

All individuals carrying *RAD50* LOF variants (Table 2) and deleterious missense variants with CADD score ≥ 25 present in familial ESCC including c.G2177A:p.R726H, c.C2287T:p.R763C, c.G3716A:p.R1239Q, and c.C3790T:p.L1264F (Table S1) were validated by Sanger sequencing, as shown in Figure 2 for representative validation of LOF variants c.C2014T:p.Q672X, and c.2165_2166insT:p.K722fs located at exon 13 containing the zinc hook domain and the missense c.C3790T:p.L1264F variant at exon 25.

### 3.4. RAD50 Is Indispensable for the Survival of ESCC Cells

**Figure 3:**
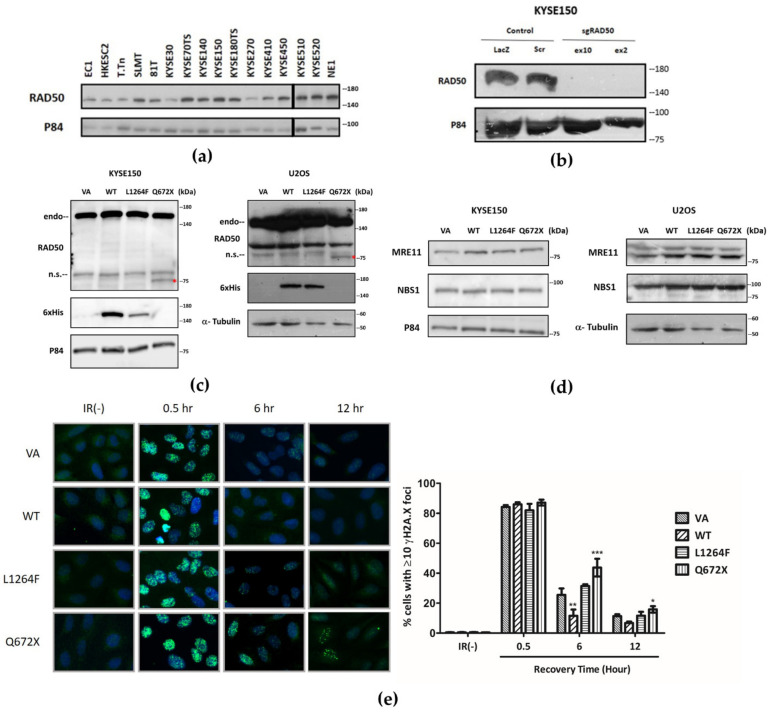
Dominant negative effect of DSB repair by overexpression of zinc hook *RAD50* ^Q672X^ mutant. RAD50 ubiquitously expressed in ESCC cell lines (**a**); knockdown of *RAD50* in KYSE150 (**b**); overexpression of *RAD50* mutants in KYSE150 and U2OS (**c**) induced higher expression of MRE11 in KYSE150 and MRE11/NBS1 in U2OS (**d**); delay of DSB repair ability indicated by γH2AX foci formation in *RAD50*^Q672X^ overexpression in U2OS after IR stimulation (**e**). *, **, *** indicate *p* < 0.05, 0.01, and 0.001, respectively. Endo: endogenous full-length RAD50, n.s.: nonspecific, Red asterisk: RAD50-Q672X.

As a gene well-documented to be essential to cellular survival, RAD50 protein is constitutively expressed in a panel of fifteen ESCC cell lines (Figure 3a). The expressions of RAD50 in ESCC cell lines KYSE150 and KYSE180TS were the highest amongst the ESCC cell lines tested. When compared to NE1, an immortalized cell line derived from normal esophageal tissue, ESCC cell lines generally showed reduced RAD50 expression levels. To study the functional role of *RAD50* in ESCC, we performed CRISPR-Cas9-mediated functional knockout of *RAD50* in ESCC cell line KYSE150. After confirmation of successful depletion of RAD50 by Western blot (Figure 3b), it was observed that KYSE150 cells lost viability rapidly and ceased to proliferate, in contrast to the controls in the knockout experiment. This observation conforms to the expected result for an essential role of *RAD50* in cell survival in an ESCC cell line.

### 3.5. Dominant Negative Overexpression of RAD50^Q672X^ Mutant Delays the Repair of IR-Induced DSBs

From our NGS data, LOF and missense germline mutations of *RAD50* that are potentially functionally disruptive were identified (Figure 1 and Table S1). In particular, mutant *RAD50*^L1264F^ and *RAD50*^Q672X^ reside in close proximity to the ATP-binding cassette and zinc hook domain of *RAD50*, respectively. Hence, we performed site-directed mutagenesis to obtain the mutated constructs and overexpressed these mutants (*RAD50*^L1264F^, *RAD50*^Q672X^) and wildtype *RAD50* (*RAD50*^WT^) *in vitro* to study the phenotypic consequences of these mutations. Upon validation of overexpression of *RAD50* constructs in both KYSE150 and U2OS by Western blot (Figure 3c), we examined the expression levels of the interacting proteins of RAD50 in the MRN complex. Overexpression of *RAD50*^WT^, *RAD50*^L1264F^, and *RAD50*^Q672X^ results in a proportional increase in the protein expression levels of MRE11 and NBS1 (Figure 3d).

The primary function of RAD50 or the MRN complex is in the initiation of DNA damage response to DSBs. Hence, we assessed the effect of mutant expression on the formation of γH2AX foci after irradiating cells with gamma-radiation for 30 min followed by 6 and 12 h of recovery post-irradiation (Figure 3e). The overexpression of both *RAD50*^WT^ and mutants did not affect the formation of γH2AX foci 30 min post-IR. Six hours after the initial IR treatment, the overexpression of *RAD50*^WT^ showed more rapid repair of IR-induced DSBs, marked by the significantly reduced percentage of γH2AX foci-positive cells. Whilst overexpression of either *RAD50*^L1264F^ or *RAD50*^Q672X^ did not enhance the recovery of γH2AX foci, *RAD50*^Q672X^ showed further hindrance in the recovery of foci, as a significantly larger percentage of foci-positive cells persisted 6 and 12 h post-IR.

### 3.6. Dominant Negative Overexpression of RAD50 Mutants Sensitize Cells to Formaldehyde and CHK1 Inhibitor AZD7762 Treatment

**Figure 4:**
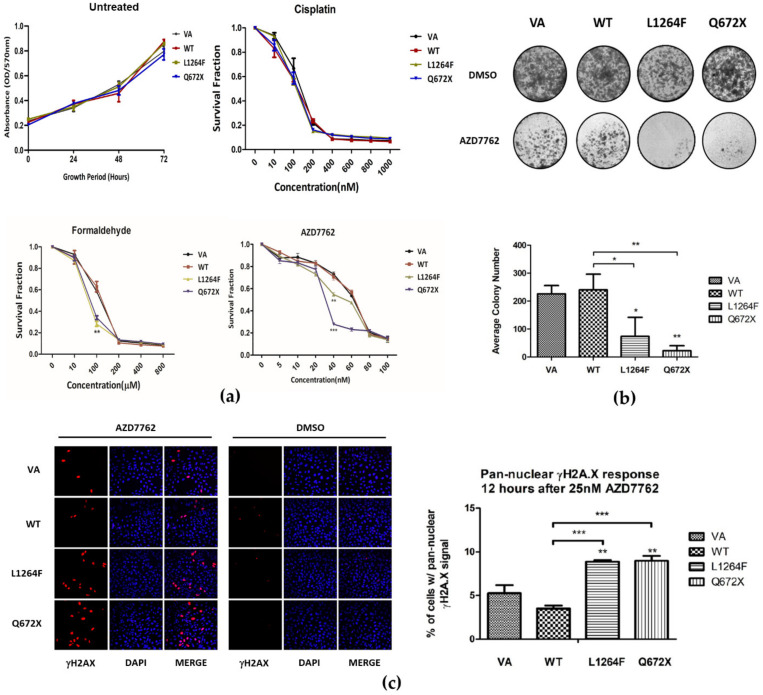
(**a**) Dominant negative effect of overexpression of *RAD50*^Q672X^ and *RAD50*^L1264F^ in U2OS sensitized cell viability to AZD7762 (CHK1 inhibitor) and formaldehyde treatment but not to cisplatin; (**b**) inhibited colony forming ability; and (**c**) increased pan-nuclear γH2A.X response after AZD7762 treatment. *, **, *** indicate *p* < 0.05, 0.01, and 0.001, respectively.

Previous studies targeting *RAD50* or the MRN complex combined with cisplatin treatment resulted in tumor cell sensitization [16,25,26]. The dominant-negative functional impact of *RAD50*^Q672X^ in delayed DSB repair upon IR treatment led us to test the sensitivity of cells expressing *RAD50* wildtype and mutant constructs with cisplatin and a PARP inhibitor (PARPi). The MTT assay indicated that the expression of *RAD50* mutants did not potentiate cells toward cisplatin (Figure 4a) and PARPi treatment (Table S2). The dominant-negative LOF *RAD50* mutant did not enhance therapeutic efficacy of platinum-based drugs. A previous study revealed *RAD50* activated *ATR* signaling upon replication stress [41]. We tested the sensitivity of cells expressing *RAD50* wildtype and mutant constructs toward formaldehyde that induces DNA damage through replication stress. The MTT results showed that *RAD50*^L1264F^ and *RAD50*^Q672X^ cells were sensitized upon formaldehyde treatment at 100 µM and toward AZD7762, an ATP-competitive and selective checkpoint kinase inhibitor (CHK1) inhibitor, with an IC_50_ of 40.72 and 28.91 nM, respectively, compared to VA control (64.53 nM) and *RAD50*^WT^ (66.49 nM) (Figure 4a). The sensitization toward AZD7762 treatment was also evidenced in the colony formation assay (Figure 4b). As for the phenotypic cause behind the observed sensitization, we observed, upon AZD7762 treatment, a significantly elevated percentage of *RAD50*^L1264F^ and *RAD50*^Q672X^ cells showed pan-nuclear γH2AX patterns, indicative of cytotoxic level of replication stress, in contrast to the vector-alone control and *RAD50*^WT^ (Figure 4c).

## 4. Discussion

Our current NGS study is the first comprehensive investigation of the entire *RAD50*, (also includes *MRE11A* and *NBS1*) by hybridization-based target capture approach for the genetic predisposition role of the evolutionarily conserved MRN complex in the high-risk ESCC region. Our earlier publication utilized a gene-based association test and revealed no significant association of elevated cancer risk in Henan familial ESCC patients carrying LOF variants in *RAD50-MRE11A-NBS1* [15]. With more detailed data analysis in the *RAD50* gene, two rare pathogenic LOF variants in the zinc hook domain of *RAD50* were identified to only be present in familial ESCC patients but were not found in the sporadic ESCC patients and controls (Table 3). These zinc hook variants, *RAD50*^Q672X^ and *RAD50*^K722fs^, were about 10-fold and 100-fold less frequent in East Asian and all populations including African, Latino, Jewish, European, South Asian, and East Asian populations in the genomAD database. The absence of zinc hook LOF germline mutations *RAD50*^Q672X^ and *RAD50*^K722fs^ in another large *RAD50* breast cancer study in 7657 Chinese patients further demonstrated their potential genetic predisposition role in familial ESCC [29]. While the significance of the two zinc hook pathogenic mutations of *RAD50* based on their incidence do not justify their use in generalized screening due to their rarity in the population-at-large observed in the current study with 4 patients out of 1044 familial ESCC cohort and the publicly available reported incidence of 4 out of 19,945 East Asian noncancer individuals; however, they are clinically important. The ClinVar database reported one breast cancer and four hereditary cancer predisposing syndrome patients carrying either *RAD50*^Q672X^ and *RAD50*^K722fs^ [39,40]. Importantly, when we consider the *RAD50* zinc hook domain localized to amino acid residues at 635–734 [28], three more zinc hook LOF pathogenic mutations (*RAD50*^S653X^ [42], *RAD50*^R656X^ [43], and *RAD50*^E676X^ [44]) were reported in one NBS-like disorder and another six hereditary cancer predisposing syndrome patients from the ClinVar database. Several other DNA repair genes including *BRCA2*, *MSH2*, and *POLQ* were previously reported to be associated with a higher risk of familial ESCC [15]. High-risk individuals from ESCC FH+ families may benefit from noninvasive genetic profiling tests targeting a sequence constellation of these DNA repair genes associated with hereditary cancer for cancer risk assessment.

Platinum-based drugs, such as cisplatin, are the common first-line chemotherapeutic drugs for ESCC. A dual disruption approach targets DNA repair and telomere maintenance to treat *BRCA*-proficient head and neck cancer by combining PARPi and dominant negative *Nbs1* disruption of MRN [45]. However, the protein truncating *RAD50*^Q672X^ mutant affecting the zinc hook and eliminating the C-terminal coiled-coil and ATPase domains, which resulted in a delay of DSB repair, did not sensitize cells upon cisplatin or PARPi treatment. The dominant negative LOF *RAD50* mutant did not enhance the therapeutic efficacy of platinum-based drugs. Interestingly, our study suggested novel insights for the potential synthetic lethal therapeutic options for zinc hook mutations and AZD7762 as the functional characterization of the zinc hook protein truncation mutant, *RAD50*^Q672X^, suggested a dominant negative effect in DSB repair and replication stress. Further functional studies are warranted for additional germline *RAD50* zinc hook LOF mutations including K722fs identified in the current study, *RAD50*^S653X^ [42], *RAD50*^R656X^ [43], and *RAD50*^E676X^ [44] from ClinVar databases as these mutants are expected to act in the dominant negative fashion upon replication stress that may have importance for a potential therapeutic option.

Mutations of the MRN complex were present in about 4% of all human tumors and clustered near the D-loop motif in various tumor types [46]. Tumors harboring a *RAD50*^L1237F^ hypomorphic mutant allele at the D-loop motif of the RAD50 protein and defective ATM signaling demonstrated an outlier curative response in a recurrent patient treated with irinotecan and AZD7762, targeting CHK1 inhibition [46]. As shown in Table S1, *RAD50*^L1264F^ is relatively more frequently detected in 4 FH+ ESCC, 4 sporadic ESCC, 3 controls in our cohort, and 80 East Asians but absent in 64517 Europeans from gnomAD compared to the zinc hook mutants. *RAD50*^L1264F^ is an East Asian-specific deleterious missense mutation with combined annotation dependent depletion (CADD) score of 30 ranked among top 0.1% predicting deleteriousness [34]. It lies within the C-terminal ATPase between D-loop and the H-loop/switch. Although the deleterious mutant *RAD50*^L1264F^ did not associate with higher familial ESCC risk, our functional study suggested patients carrying this East-Asian-specific heterozygous *RAD50*^L1264F^ (Table S1) may have a dominant negative effect upon induction of replication stress by formaldehyde treatment and the CHK1 inhibitor, AZD7762.

The functional study further reinforced our NGS association of the elevated risk of germline LOF of *RAD50* in the zinc hook domain with familial ESCC. The large effect of the observed zinc hook LOF mutations with familial ESCC may be overestimated due to their rarity and limitations by our study with a moderate sample size.

## 5. Conclusions

Our study is the first to suggest the association of two rare *RAD50* zinc hook LOF mutations with familial ESCC in Northern Chinese and implicate the potential genetic predisposition role of the *RAD50* zinc hook domain in ESCC genetic pathogenesis. Our results suggest that the dominant negative effects of the *RAD50*^Q672X^ mutant impair cellular responses to DSB repair and enhance sensitivity to genotoxic agents including checkpoint inhibitor AZD7762 and formaldehyde-induced replication stress.

## Figures and Tables

**Table 1:** Details of study populations.

Populations	Phase	Definition	Number of Individuals	Number of Alleles (Number of Individuals × 2)
Family history-positive (FH+) ESCC	Discovery	ESCC patients with two generations and ≥2 family members diagnosed with ESCC including proband from Henan	186	372
FH+ ESCC	Validation	ESCC patients with one generation and ≥2 family members diagnosed with ESCC including proband from Henan	858	1716
FH+ ESCC	Combined discovery and validation	ESCC patients with one or two generations and ≥2 family members diagnosed with ESCC including proband from Henan	1044	2088
Sporadic ESCC	Validation	ESCC patients without known history of ESCC in the family members from Henan	1074	2148
Control	Validation	Non-ESCC individuals from Henan	1171	2342
Non-FH+ control population	Validation	Combined sporadic ESCC and control populations	2245	4490
gnomAD East Asian	-	East Asian population from gnomAD, https://gnomad.broadinstitute.org/ accessed on 5 June 2020	9977	19,954
gnomAD All	-	All populations from gnomAD including African, Latino, Jewish, European, East and South Asian https://gnomad.broadinstitute.org/ accessed on 5 June 2020	141,335	282,670

**Table 2:** *RAD50* LOF mutations identified in familial Chinese ESCC patients, sporadic ESCC, and controls in 3289 individuals from Henan.

Chr5 (hg19)	Mutation (DNA) ^a^	Exon	Protein Change	Familial ESCC Cases (1044) Discovery FH+ (186) (*n* = 372) Validation FH+ (858) (*n* = 1716)	Validation Sporadic ESCC Cases (1074) (*n* = 2148)	Validation Controls (1177) (*n* = 2342)	gnomAD ^b^ East Asian (9977) (*n* = 19954)	*p*	OR
**Whole-exome sequencing of discovery cohort of 186 FH+ ESCC involved two generations**									
131,931,309	c.C2014T	13	p.Q672X	1	NA	NA	0		
131,931,460	c.2165_2166insT	13	p.K722fs	1	NA	NA	4		
131,945,032	c.2980_2983del	19	p.K994fs	1	NA	NA	8		
131,951,735	c.3077_3080del	20	p.K1026fs	1	NA	NA	Not reported		
	Total Discovery			4/372			30/19954	**3.3 × 10^−3 c^**	7.22 ^c^
**Target whole-gene sequencing of *RAD50* in validation cohort of 3103 individuals containing 858 FH+ and 1074 sporadic ESCC and 1171 controls**									
131,924,437	c.1110delA	8	p.L370fs	1	2	2	Not reported ^g^	0.37 ^d^	1.97 ^d^
13,193,1460 [28]	c.2165_2166insT	13	p.K722fs	2	0	0	4		
131,944,319	c.C2731T	17	p.Q911X	0	0	1	Not reported	0.38 ^e^	1.80 ^e^
131,945,032	c.2980_2983del	19	p.K994fs	0	1	1	8		
131,973,850	c.C3553T	23	p.R1185X	0	1	0	1	1.00 ^f^	1.09 ^f^
		Combined Total		7/2088	4/2148	4/2342			

^a^ Nucleotide position are based on the NM_005732 transcript of *RAD50*. ^b^ Frequencies of *RAD50* variants are exported from gnomAD, https://gnomad.broadinstitute.org/, (accessed on 5 June 2020). ^c^ Fisher exact test compared frequency of *RAD50* LOF variants (4/372) from FH+ Chinese ESCC with that (30/19954) from East Asian population from gnomAD Exomes (exclude p.L719fs at chr5: 131931451) in the discovery cohort. *n* = number of alleles. Bolded value indicates statistically significant association. ^d^ Fisher exact test compared frequency of *RAD50* LOF gain variants (7/2088) from familial Chinese ESCC combining discovery and validation cohorts with that (4/2342) from Henan controls. ^e^ Fisher exact test compared frequency of *RAD50* LOF variants (7/2088) from familial Chinese ESCC combining discovery and validation cohorts with that (4/2148) from sporadic ESCC. ^f^ Fisher exact test compared frequency of *RAD50* LOF variants (4/2148) from sporadic ESCC with that (4/2342) from controls. ^g^ p.L370fs reported with frequency of 1/15314 in a *RAD50* association study for Chinese breast cancer patients (*n* = 7657) [29]. Bolded value indicates statistically significant association.

**Table 3:** Estimated risk of familial ESCC with LOF *RAD50* variants in Henan cohort of familial ESCC versus sporadic ESCC and controls, and East Asian population or all populations from gnomAD.

Mutation	Familial ESCC (*n* = 2088)	Sporadic ESCC (*n* = 2148)	Controls (*n* = 2342)	*p* ^a^	OR ^a^	gnomAD ^d^ East Asian (*n* = 19,954)	*p* ^b^	OR ^b^	gnomAD ^d^ All (*n* = 282,670)	*p* ^c^	OR ^c^
p.Q672X	0.045% (1)	0%	0%	0.32	inf	0%	0.095	inf	0.00035% (1)	**0.015**	135.44
p.K722fs	0.14% (3)	0%	0%	**0.032**	inf	0.02% (4)	**0.022**	7.18	0.0014% (4)	**1.3 × 10^−5^**	101.73
p.Q672X/ p.K722fs	0.19% (4)	0%	0%	**0.010**	inf	0.02% (4)	**4.1 × 10^−3^**	9.57	0.0018% (5)	**3.5 × 10^−7^**	108.51
All *RAD50* LOF	0.34% (7)	0.19% (4)	0.17% (4)	0.33	1.88	0.15% (30)	0.092	2.23	0.17% (427)	0.062	2.22

^a^*p* value and OR of Fisher exact test comparing frequency of *RAD50* zinc hook mutations from Henan Chinese familial ESCC with that from sporadic ESCC and controls (0/4490). pL719fs was filtered. ^b^ *p* value and OR of Fisher exact test comparing frequency of *RAD50* zinc hook mutations from Henan Chinese familial ESCC with that from East Asian population from gnomAD. ^c^ *p* value and OR of Fisher Exact test comparing frequency of *RAD50* zinc hook mutations from Henan Chinese familial ESCC with that from all populations from gnomAD. ^d^ Frequencies of *RAD50* variants are exported from gnomAD, https://gnomad.broadinstitute.org/ (accessed on 5 June 2020). Bolded values indicate statistically significant association.

## Data Availability

Sequence data were deposited at the European Genome-phenome Archive (EGA), accession number EGAS00001003423. The data that support the findings of our study are available from the corresponding author upon reasonable request.

## Supplementary Materials

The following are available online at https://www.mdpi.com/article/10.3390/cancers13184715/s1, Table S1: Deleterious missense *RAD50* variants with CADD score ≥ 25. Table S2: The IC_50_ of various genotoxic compounds in U2OS overexpressing vector alone (VA), *RAD50*^WT^, *RAD50*^Q672X^, and *RAD50*^L1264F^. Figure S1: Original western blots.
