# Supplementary material for: RAD50 Loss of Function Variants in the Zinc Hook Domain Associated with Higher Risk of Familial Esophageal Squamous Cell Carcinoma
Source: Cancers (Basel). 2021 Sep 21;13(18):4715. doi: 10.3390/cancers13184715 (PMC8472384; doi:10.3390/cancers13184715)
Supplement: Supplementary file 1 [file cancers-13-04715-s001.zip › cancers-1342015-original-images -revised.pdf]

Figure S1a.

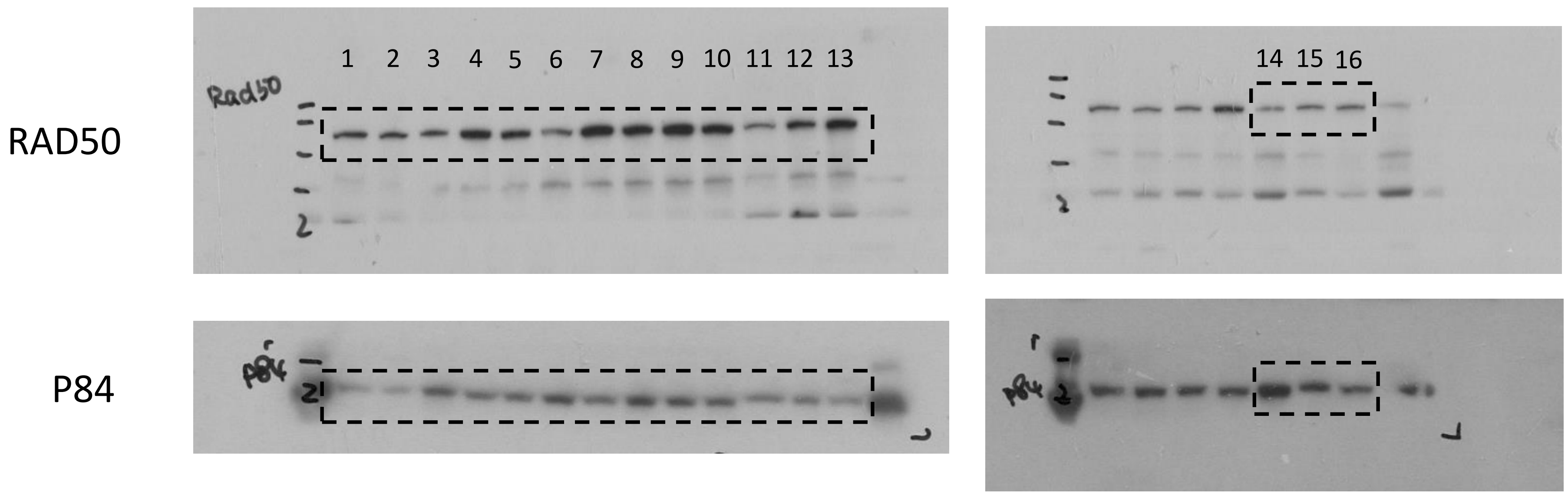

Cell lines:

1. **EC1**
2. **HKESC2**
3. **T.Tn**
4. **SLMT**
5. **81T**
6. **KYSE30**
7. **KYSE70TS**
8. **KYSE140**
9. **KYSE150**
10. **KYSE180TS**
11. **KYSE270**
12. **KYSE410**
13. **KYSE450**
14. **KYSE510**
15. **KYSE520**
16. **NE1**

Figure S1b.

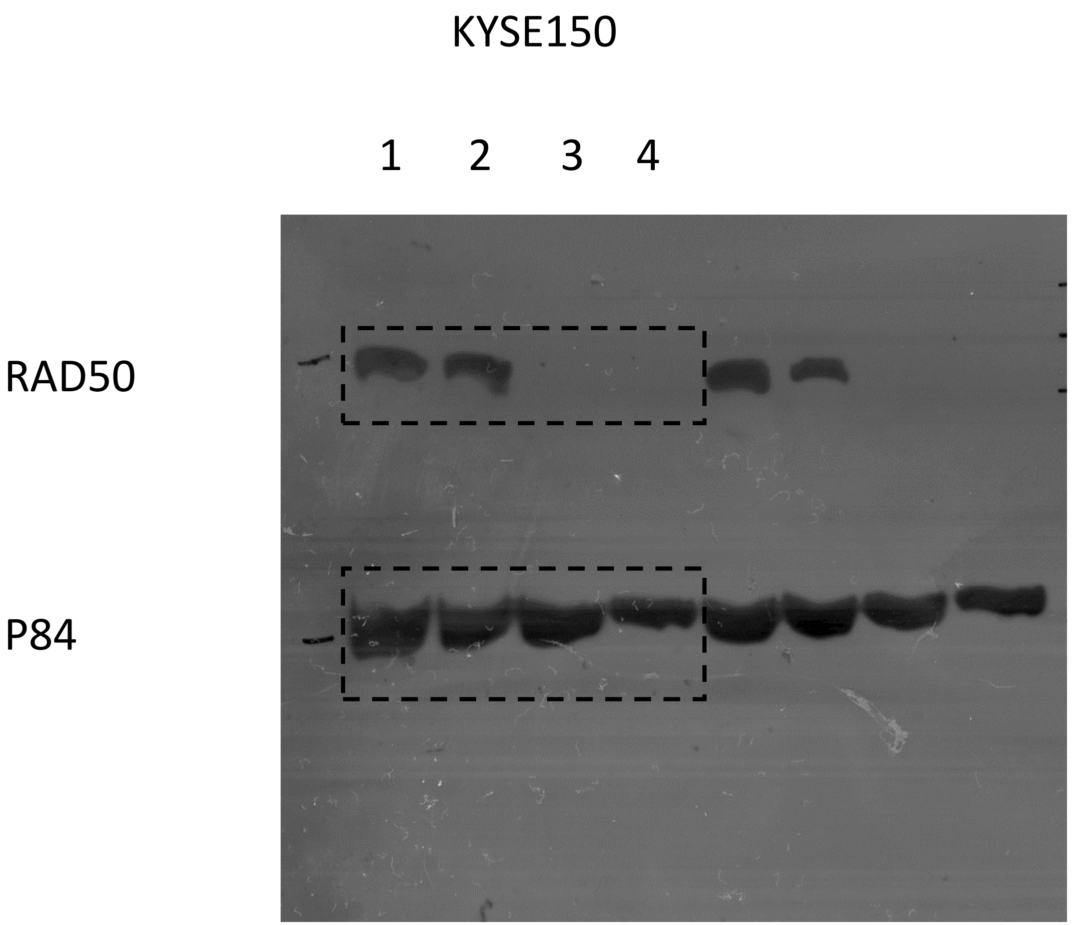

1: LacZ Control. 2: Scramble Control. 3: sgRAD50 ex10. 4. sgRAD50 ex2

Figure S1c.

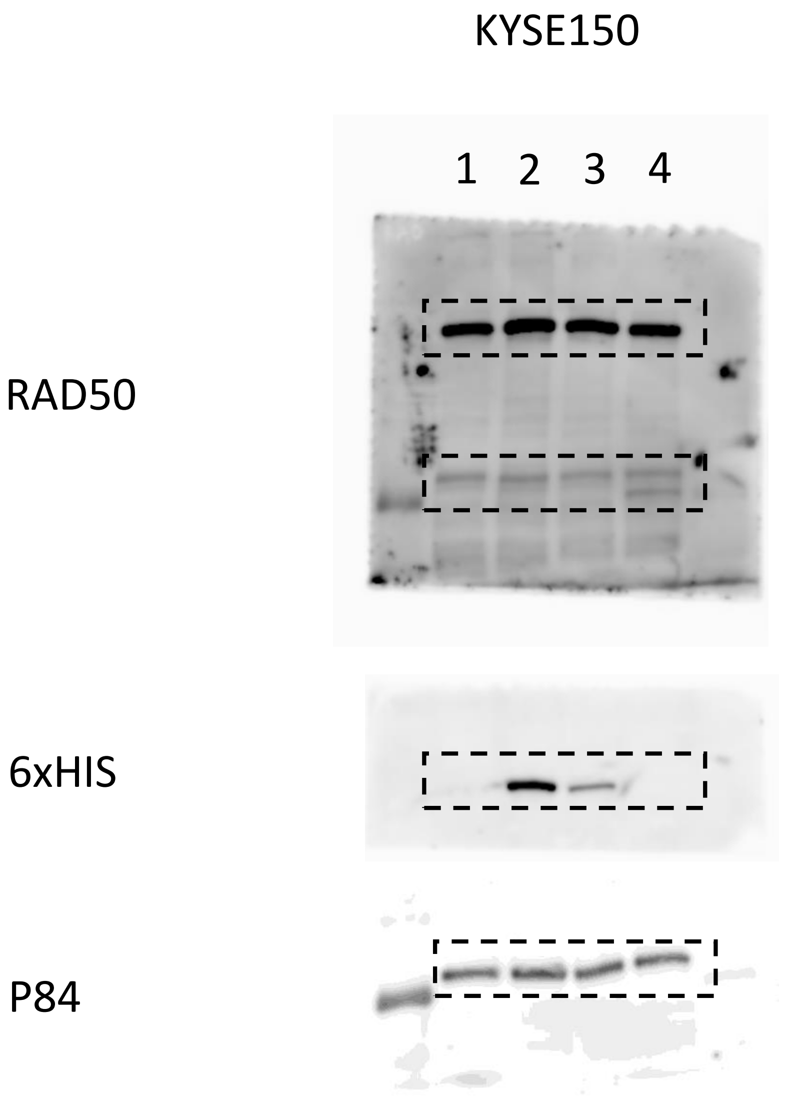

1: Vector-alone control. 2: RAD50-Wildtype. 3 RAD50-L1264F. 4. RAD50-Q672X

Figure S1c.

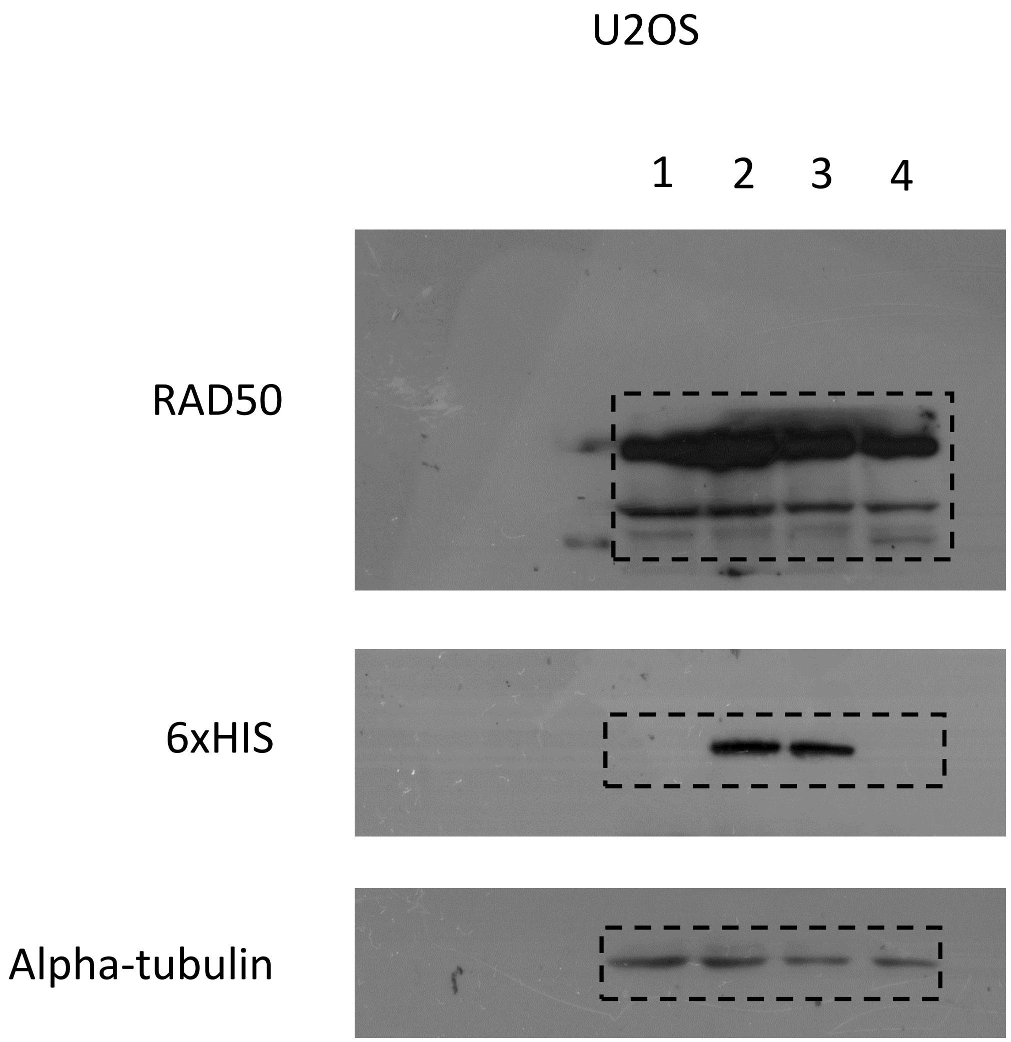

Image of lighter exposure of RAD50

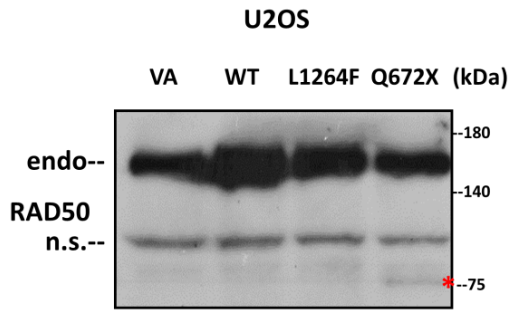

1: Vector-alone control. 2: RAD50-Wildtype. 3: RAD50-L1264F. 4. RAD50-Q672X
Note: U2OS samples in Figure 3c and 3d belongs to the same lysate samples run on the same SDS—PAGE gel, hence shared same loading control (Alpha-tubulin)

Figure S1d.

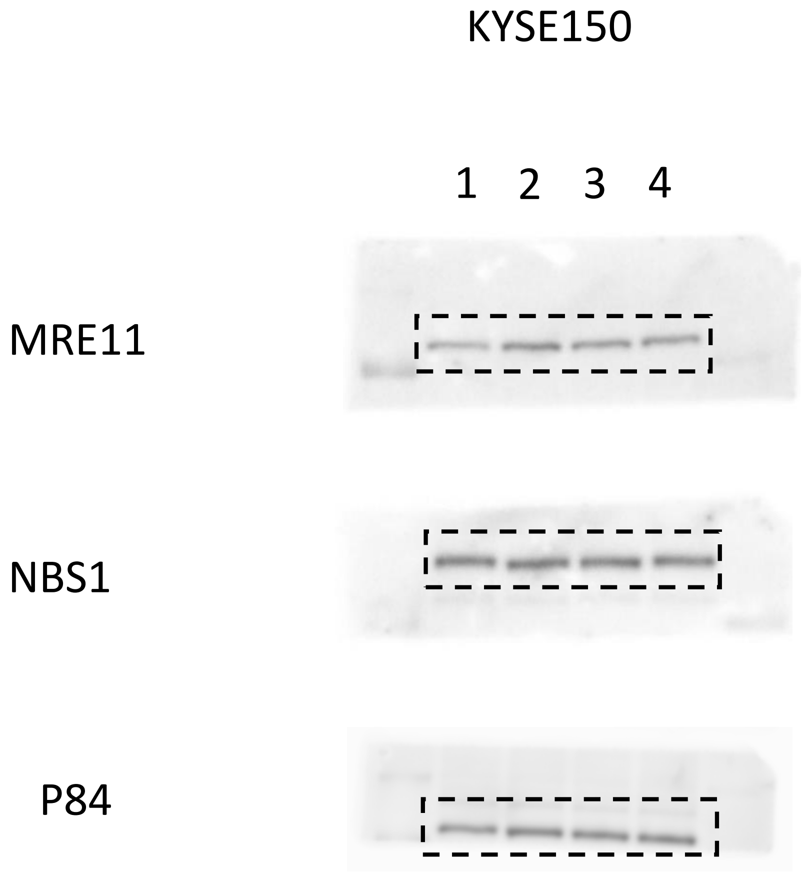

1: Vector-alone control. 2: RAD50-Wildtype. 3 RAD50-L1264F. 4. RAD50-Q672X

Figure S1d.

U2OS

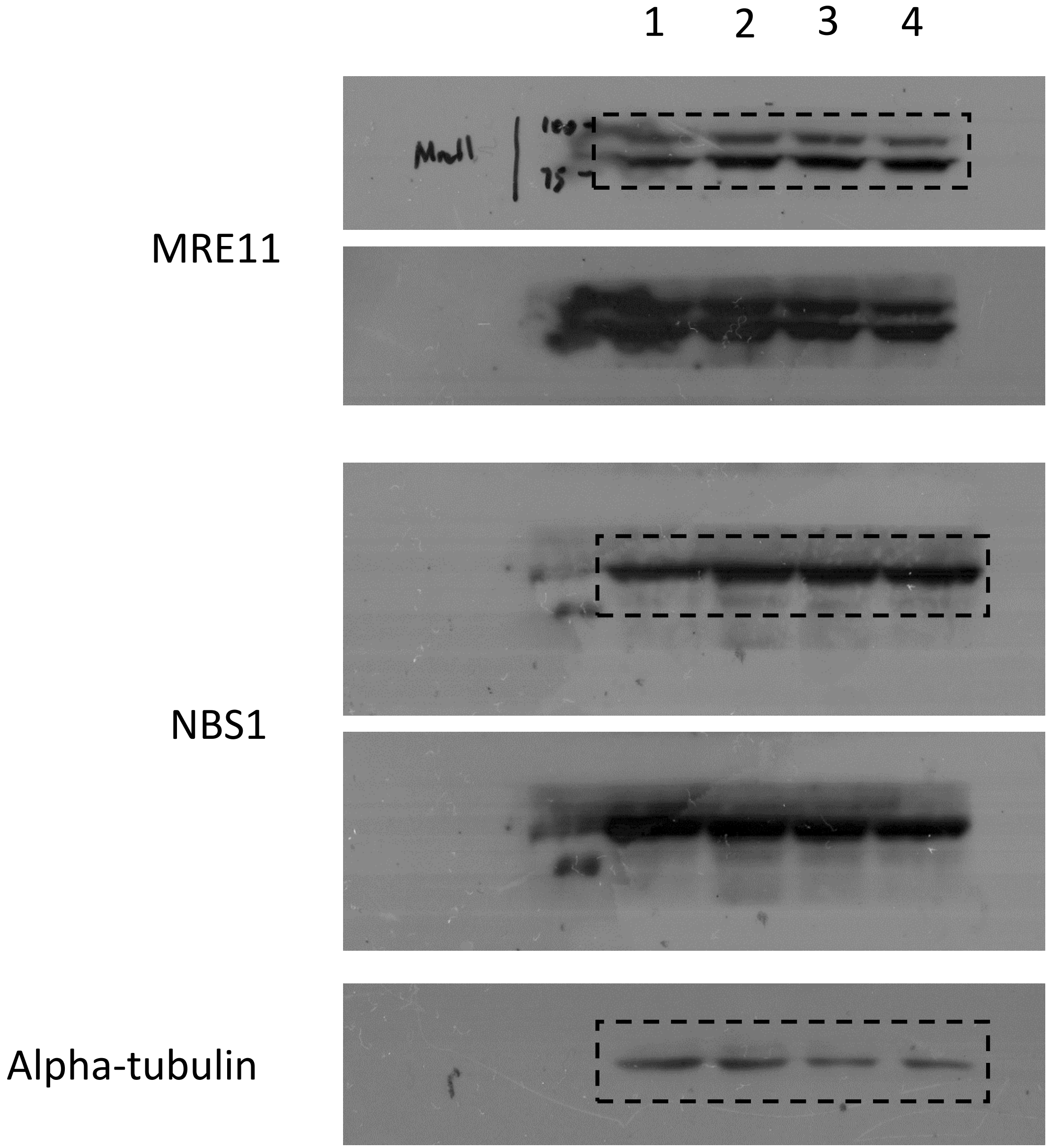

1: Vector-alone control. 2: RAD50-Wildtype. 3: RAD50-L1264F. 4. RAD50-Q672X

Note: U2OS samples in Figure 3c and 3d belongs to the same lysate samples run on the same SDS—PAGE gel, hence shared same loading control (Alpha-tubulin)
