# Supplementary material for: RAD50 Loss of Function Variants in the Zinc Hook Domain Associated with Higher Risk of Familial Esophageal Squamous Cell Carcinoma
Source: Cancers (Basel). 2021 Sep 21;13(18):4715. doi: 10.3390/cancers13184715 (PMC8472384; doi:10.3390/cancers13184715)
Supplement: Supplementary file 1 [file cancers-13-04715-s001.zip › cancers-1342015-Table-supplementary.pdf]

Table S1: Deleterious missense *RAD50* variants with CADD score >=25

**Chr5 (hg19)**	**Mutation (DNA)[a]**	**CADD 1.4**	**Exon**	**Protein change**	**FH+ cases (n=2088)**	**Sporadic cases (n=2148)**	**Controls (n=2342)**	**gnomAD[b]**		
								**East Asian (n=19954)**	**European (n=129034)**	**All (n=282716)**
131911608	c.T353C	27.7	3	p.I118T	0	0	2 (8.5x10^{-4})	17 (9.3x10^{-4})	0	17 (6.0x 0^{-5})
131931472	c.G2177A	25.8	13	p.R726H	1 (4.8x10^{-4})	0	0	0	15 (1.2x10^{-4})	30 (1.1x10^{-4})
131939071	c.C2287T	25.2	14	p.R763C	1 (4.8x10^{-4})	0	0	0	1 (8.8x10^{-6})	2 (7.0x10^{-6})
131976391	c.G3646T	32	24	p.A1216S	0	1 (4.7x10^{-4})	0	Not reported	Not Reported	Not Reported
131976461	c.G3716A	34	24	p.R1239Q	1 (4.8x10^{-4})	0	0	1 (5.4x10^{-5})	0	7 (2.5x10^{-5})
131977907	c.C3790T	30	25	p.L1264F	4 (1.9x10^{-3})	4 (1.9x10^{-3})	3 (1.3x10^{-3})	80 (4.0x10^{-3})	0	81 (2.9x10^{-4})

n is the number of alleles.

Table S2: The IC_{50} of various genotoxic compounds in U2OS overexpressing vector alone (VA), *RAD50*^{WT}, *RAD50*^{Q672X} and *RAD50*^{L1264F}.

	IC_{50}			
	**VA**	**WT**	**L1264F**	**Q672X**
**Cisplatin (nM)**	**125.6 (117.0-134.8)**	**115.4(108.9-122.4)**	**116.2(105.2-130.5)**	**115.5(106.5-125.4)**
**Olaparib(nM)**	**100.2(98.1-103.6)**	**105.2(103.2-108.6)**	**103.8(99.8-107.9)**	**100.1(95.2-104.7)**
**AZD7762 (nM)**	**64.53(55.47-75.07)**	**66.49(51.86-85.26)**	**40.72(37.8-44.2)**	**28.91(26.01-32.13)**
**Formaldehyde(uM)**	**107.6 (105.1-110.2)**	**113.1 (103.0-124.2)**	**80.12 (73.47-87.37)**	**76.47 (68.03- 85.96)**
